# Development of Multifunctional Nano-Graphene-Grafted Polyester to Enhance Thermal Insulation and Performance of Modified Polyesters

**DOI:** 10.3390/polym14183821

**Published:** 2022-09-13

**Authors:** Shih-Hsiung Chen, Naveed Ahmad, Chung-Feng Jeffrey Kuo

**Affiliations:** Department of Materials Science and Engineering, College of Engineering, National Taiwan University of Science and Technology, Taipei 10607, Taiwan

**Keywords:** polyester fabric, far-infrared rays, antistatic, Taguchi method, grey relational analysis

## Abstract

Nano-graphene materials have improved many thermal properties based on polymer systems. The additive polymers’ thermal insulation cannot be significantly increased for use as a reinforcement in multifunctional thermally insulating polymer foam. Herein, we present the development of far-infrared emissivity and antistatic properties using multifunctional nano-graphene polyester fibers. Nano-graphene far-infrared thermal insulation polyester was synthesized with 2% nano-graphene and dispersant polypropylene wax-maleic anhydride (PP wax-MA) using the Taguchi method combined with grey relational analysis (GRA) to improve the thermal properties and the performance of the polymer composite. The thermogravimetric analysis (TGA) shows that the pyrolysis temperature of spinning-grade polyester was increased when the nano-graphene powder was added to the polyester. The differential scanning calorimeter (DSC) analysis confirmed the modification of polyester by nano-graphene, showing the effect of the nucleating agent, which ultimately improved the performance of the polyester. The physical properties of the optimized polyester fibers were improved with a yarn count of 76.5 d, tensile strength of 3.3 g/d, and an elongation at break increased from 23.5% to 26.7% compared with unmodified polymer yarn. These far-infrared emission rates increased from 78% to 83%, whereas the far-infrared temperature increased from 4.0 °C to 22 °C, and the surface resistance increased to 10^8^ Ω. The performance of the optimized modified polyester yarn is far better than single-polypropylene-grafted maleic anhydride yarn. The performance of optimized modified polyester yarn, further confirmed using grey correlation analysis (GRA), can improve the yarns’ mechanical properties and far-infrared functions. Our findings provide an alternative route for developing nano-graphene polyester fabrics suitable for the fabric industry.

## 1. Introduction

Natural fibers have been used as the primary materials because of thermal insulation, do not produce poisonous gases on burning, and are readily available. Natural fibers manufacture comfortable cloths; however, natural fibers’ strength is insufficient. They are heavyweight, can be damaged by insects, are not wrinkle-free, and are not long-lasting; thus, they are not durable for longer. Therefore, in the last few decades, synthetic fibers have been developed and increasingly used for modified fabric and thermal insulation products. Lin et al. [1] described how synthetic fibers related to textile materials’ thermal insulation are either passive or active. These types are able to keep warm by controlling the content of the still air layer, such as hollow fibers, and heating the human body to achieve the effect of heat preservation by adding a heating device. Conventional heat-generating fibers keep the body warm through the enhancement of thermal insulation. For instance, a hollow fiber has been applied to the air trap to retain warm air and enhance insulation. Even though the fiber is wet, showing good thermal insulation, there is a limit in keeping warm since this kind of fiber only has a thermal insulation effect without generating heat [2,3]. The bamboo charcoal also shows thermal insulation properties, and this powder is often added to fibers, which can then be used for different wearing items [1]. Photothermal conversion composites based on textile materials have attracted extensive attention in various fields such as smart textiles, photothermal therapy, and heat preservation [4,5,6]. With the recent development of functional textile materials which enhance health, there is an increasing demand for textiles with even more excellent benefits to body health. For instance, far-infrared radiation is an essential characteristic of restorative materials. Using “healthy fibers” to manufacture functional textiles with far-infrared emission properties has become a significant research direction in functional materials [2].

Kim et al. [7] reported the far-infrared emission properties and wearing comfort of embedded ZrC PET (polyethylene terephthalate) heat-storage knitted fabrics for sentimental garments. The thermal and drying properties of the ZrC-imbedded PET knitted fabric were measured and compared with those of the regular PET knitted fabric. The mechanical properties using the FAST (fabric assurance by simple testing) system and the dye affinity of the ZrC-embedded knitted fabric were compared with those of regular PET knitted fabric. Lin et al. [1] examined thermal insulation by far-infrared emission characteristics using carbonized-powder-treated nonwoven charcoal fibers [8] and developed a heat storage fabric using 2 wt% ZrC-embedded in PET and confirmed the increased moisture permeability of these ZrC-embedded PET fabrics. Bahng et al. [2] developed heat-generating polyester fibers by combining heat-generating microorganisms and ceramic powder in PET. New fabric materials showed superior thermal properties for good thermal insulation compared to standard fabrics.

Moreover, ceramic powder is embedded in yarns, and the heat-generating function of fiber was found to operate normally despite many washes. This proceeding may open up a new possibility for developing functional textiles. It is also reported that the ceramic-embedded fabric has better hygroscopicity and quick-drying properties. Kim et al. [9] investigated the far-infrared emission characteristics of germanium-inlaid fabrics. They found that the emission power of germanium-inlaid fabrics had a 5–20 µm wavelength range, an intensity of 3.53 × 10^2^ W/m^2^, and an emissivity power of 0.874 W/cm^2^ microns. Improved thermal insulation performance of nylon fabrics by inserting a ceramic-embedded resin coating was also reported [10,11]. Far I.R. emissivity power was measured at a temperature of 50 °C at 7–14 µm. It showed a difference between ceramic and ceramic-coated fabrics using the thermography technique that the surface of modified fiber was increased when the fabric was heated with light. Its increased contents of ceramics ultimately enhanced the heat storage properties. Therefore, thermal insulation value increased because of increasing heat storage and infrared reflectivity. The mixed proportions of nano-bamboo charcoal materials into polyvinyl alcohol to make bamboo charcoal/PVA fibers explained the far-infrared emission characteristics of the fibers [12]. The development of three-dimensional elastic warp knitted fabrics with bamboo charcoal fibers, phase change materials, and stainless-steel fibers, reported by Huang et al. [13], could improve the fabrics’ mechanical properties and far-infrared emissivity. Adding ceramic powder to fibers makes clothes with good thermal properties, thermal insulation, and water vapor permeability properties were also studied [14].

To enhance the thermal conductivity of the polymers, the researchers investigated textiles coated with graphene or graphene derivatives. The combination of graphene with polymer exhibits various applications because graphene has unique properties such as good electrical conductivity, chemical stability, and high surface area. The graphene oxide/reduced graphene oxide nanomaterials also have significant applications such as nanosensors, optoelectronic devices, and electrochemical devices [15,16,17,18,19]. The graphene-oxide-coated fabrics for thermal conductivity purposes were also reported [20]. The wet coating technique to coat cotton fabrics using graphene nanoribbon was described by Gan et al. [21]. They explained that the graphene nanoribbon was uniformly distributed on the surface of the cotton fibers and interacted with the cotton fibers through hydrogen interactions, making it highly conductive. The graphene nanoribbon coating improves the thermal stability of cotton fabrics and tensile stress, and Young’s modulus increased by 58.9% and 64.1%, respectively. Hu et al. [22] proposed graphene/polyurethane-coated multifunctional cotton fabrics with far-infrared emission properties.

Graphene is a 2D allotrope of carbon formed from a single layer of carbon atoms bonded by sp^2^ orbitals into a hexagonal 2D lattice [23,24]. It has many good features; for example, its mechanical strength is more than 100 times that of steel, whereas its specific gravity is only about a quarter of that of steel. Its resistance is lower than copper and silver. There is currently no known material with lower resistance. Its thermal conductivity of 5300 W/mK is the highest of any known material. Its far-infrared emissivity reaches 0.97, close to the theoretical upper limit of 1 [25]. With the advancement of graphene functionalization and industrialization, graphene is now widely used in the textile industry. The so-called graphene textile refers to the effective combination of graphene and other ordinary textile materials with one or more unique properties of graphene to maintain the essential characteristics of fabrics and add more functional and intelligent features to clothing, such as far- and near-infrared health care, antibacterial, and smart textiles [26,27,28].

Therefore, this study attempts to incorporate nano-graphene powders into polyester materials to improve the heat resistance of the developed polyester fibers. The 75 d/72 f processed filament was made by melt spinning, and the nano-graphene polyester fiber with thermal insulation and far-infrared properties was obtained. Using the Taguchi method combined with GRA, six process parameters were implemented and tested, including the addition ratio of graphene powder, mold temperature, gear pump speed, melting temperature, roller speed, and take-up speed. The best single-quality process parameter values corresponding to the best multi-quality feature sets such as fiber yarn count, tensile strength, elongation at break, far-infrared radiation emissivity, and far-infrared temperature rise were obtained.

## 2. Materials and Methods

### 2.1. Materials

(1) Spinning grade PET

The source of spinning-grade PET used in this experiment is from Far east new century Co., Ltd., Hsinchu, Taiwan. The material melting point is 251.5 °C, IV viscosity = 0.65.

(2) Polypropylene wax-maleic anhydride (PP wax-MA)

The type, Licocene PP MA 6452 fine grain, is from Union Chemical Ind. Co., Ltd., Taoyuan, Taiwan.

(3) Nanographene powder

The graphene D90 powder particle size is less than 5 nm. It is from Avient Taiwan Co., Ltd., Taoyuan, Taiwan.

### 2.2. Quality Test Method

(1) Yarn count was used to measure the denier of fibers through ASTM D1577-7.
(2) The tensile strength and elongation at the break of fibers were measured by ASTM D3822.
(3) The far-infrared emissivity and far-infrared temperature rise of fabrics were measured through the FTTS-FA-010 testing standard.

### 2.3. Material Processing Process

For the graphene thermal insulation polyester fiber manufacturing process and processing equipment in this study, the manufacturing process is as follows:

#### 2.3.1. Compounding Process

**Figure 1:**
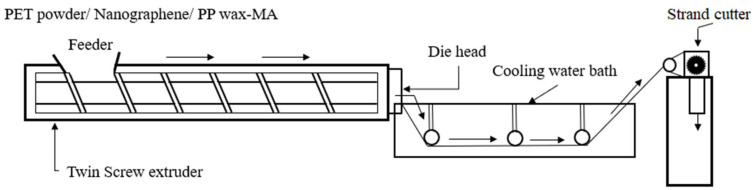
The processing diagram of a twin-screw extruder.

The twin-screw extruder is from The Japan steel works, Ltd., Osaka, Japan. It uses the interaction of two screws to replace the melts with each other to achieve the purpose of uniform mixing [29,30,31], as shown in Figure 1. In this study, PET and graphene were ground and melted in a PP wax-MA dispersant by a “powder-to-powder” approach to improve the properties of graphene and polyester for mixing uniformity. During the mixing process of PET materials, the molecular viscosity in the molten state is very high, and the diffusion rate between molecules is extremely low. There is no eddy diffusion and molecular diffusion during mixing; thus, shear force is a key factor in the polymer compounding process.

The PET composite material mixing process is as follows:

(1) Feeder: The material is fed into the feed trough.
(2) Heater: The molten material is mixed here, and the equipment used in this study has a total of thirteen sections of heaters.
(3) Die discharge port: The molten material is extruded from this, and the discharge port of the equipment used in this study is set to be a round-shaped section.
(4) Cooling system: After mixing, the material is cooled down by a water-cooling channel for cutting.
(5) Grain end: The material is cut into a granular ester state.

#### 2.3.2. Melt Spinning Process

**Figure 2:**
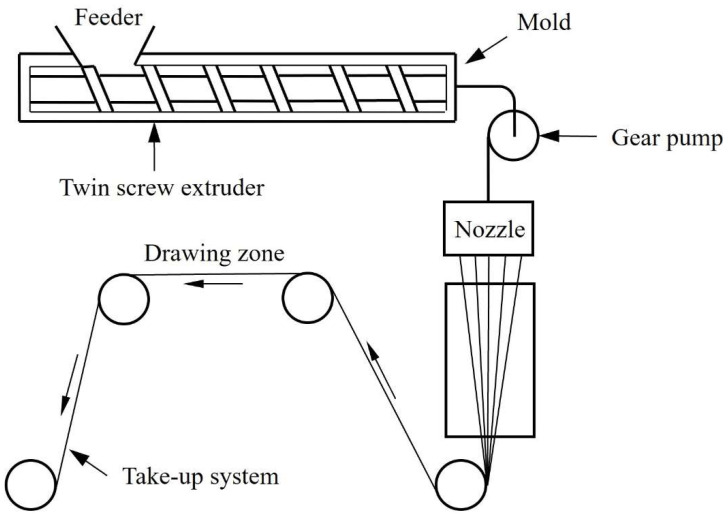
The melt spinning process diagram.

The TMT spinning system for FDY TMT Machinery from Japan is used for the melt spinning process, as shown in Figure 2 [32,33]. First, heat is used to convert the material into a molten state. It is then conveyed by a single screw extruder and metering pump to a spinning nozzle through which the multi-filaments are continuously extruded. The multi-filaments are then drawn and wound through sets of heated rolls and then wound into a filament cake. Typical melt spinning process equipment includes: feed hopper, single screw heating zone, metering pump, spinning nozzle and take-up system.

The fiber is extruded from the spinning nozzle and the multifilament processing. The processing steps are as follows:

(1) The material is propelled to the spinning nozzle through the action of a single screw and a gear pump.
(2) After the fiber is extruded from the spinning nozzle, it is still in a molten state, and the molecular chain of the fiber is produced due to the relaxation of the internal stress of the tangled molecule.
(3) Through the cooling system, solidification, and oiling and coiling system, the fibers are cooled and extended to place the fibers in the right direction, thereby increasing the strength of the fiber.
(4) The fibers are solidified and coiled into a long filament cake.

#### 2.3.3. The Chemical Reactions Taking Place during the Synthesis of the Polymer Composite

**Figure 3:**
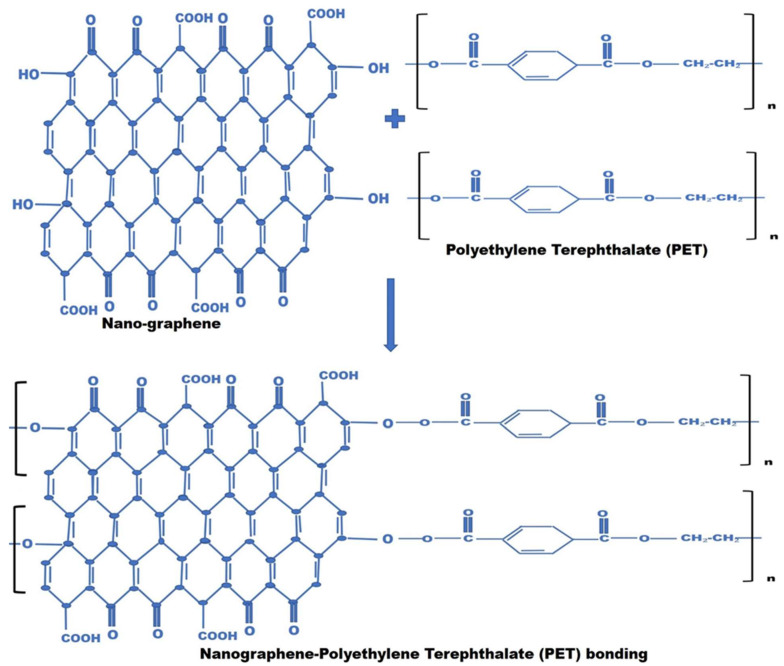

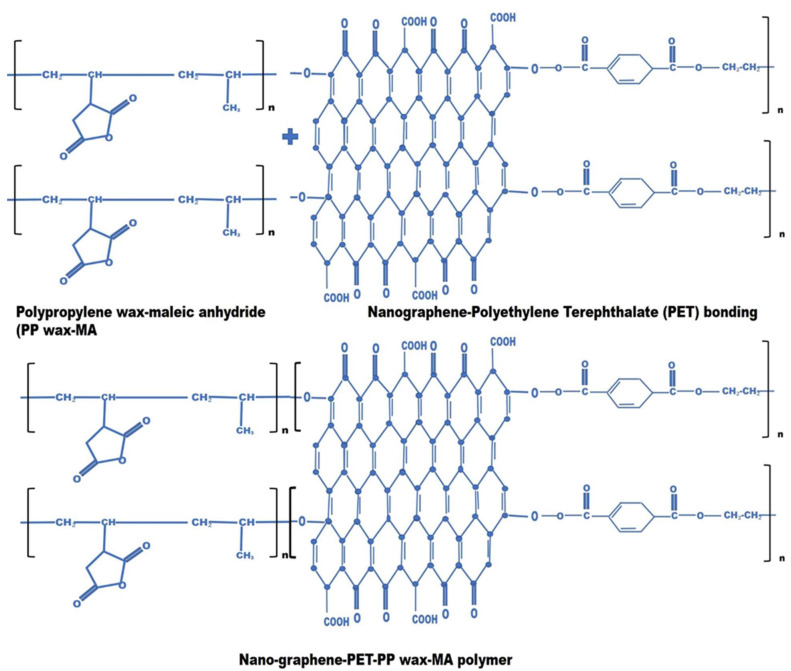
Ternary composite of nanographene-polyethylene terephthalate-polypropylene polymer.

The ternary polymer composites were prepared using the Taguchi method with PET, nanographene, PP wax-MA materials and the reactions pathway is shown in Figure 3. In this reaction, nano-graphene sheets bonded to the PET molecule, to synthesize large linear and cross-linking polymers. The condensation reaction occurs during the formation of large linear polymer of graphene-PET with the elimination of small molecules such as H_2_O, etc., when nano-sized graphene reacted with PET. After the synthesis of nano-graphene-PET composite, this composite is reacted with another polymer PP wax-MA wax, which resulted in a linear linkage nano-graphene-PET-PP wax-MA wax ternary polymer composite. In the first step of the reaction, ester linkage is formed between nanographene, and PET; however, when PP wax-MA is reacted in the second step of the reaction, ether linkage is formed. Therefore, ester and ether linkage occurred during the synthesis of the polymer.

Here, an important point is noted, namely, that when polymer is bonded to graphene, it faces π–π stacking, which ultimately leads to an increase in crystallinity and improved resistance for the hydrolysis. The resulting composite material shows unique mechanical and physical properties.

## 3. Process Optimization

### 3.1. Taguchi Quality Method

The Taguchi quality method [34,35,36,37] uses orthogonal arrays to carry out experiments and employs signal-to-noise (S/N) ratios to analyze the corresponding experiment data.

(1) Orthogonal array

The orthogonal array used in this study is L_18_(3^6^). There are 18 experiments and 6 tri-levels.

(2) S/N ratio

The yarn count, tensile strength, elongation at break, far-infrared emissivity, and far-infrared temperature rise are expected to be obtained as maximum. The quality characteristics are Larger-the-Better. The S/N ratio is:

(1) $$S/N\;\mathrm{ratio}=-10\;\log\;(\frac{1}{n}\sum_{i=1}^{n}\frac{1}{y_{i}^{2}})$$

where y_i_ is the measured value of quality i, and n is the total number of measurements.

(3) Main effects analysis (MEA)

The larger the main effect value of a factor will be the greater the influence of that factor on the system compared to other factors and vice versa.

(2) $${\bar{F}}_{i}=\frac{1}{m}\sum_{j=1}^{m}y_{i}$$

(3) $$\Delta F=\max\left\{{\bar{F}}_{1},{\bar{F}}_{2},\ldots ,{\bar{F}}_{n}\right\}-\min\left\{{\bar{F}}_{1},{\bar{F}}_{2},\ldots ,{\bar{F}}_{n}\right\}$$

where ${\bar{F}}_{i}$ is the average response value of the various factor levels, n is the factor level, m is the number of level i observations in the factor column of the orthogonal array, and $y_{i}$ is the S/N ratio of each i level row.

### 3.2. Analysis of Variance (ANOVA)

The S/N ratio obtained from the Taguchi experimental design is used to demonstrate the impact of factors on the overall experiments. The ANOVA [32,33,34,35] is described as follows:

(1) Degrees of freedom (DOF):

The DOF of a single factor equals its level number minus one. The total DOF is the total number of experiments minus one. The error of DOF (DOF_E_) is the total DOF minus the sum of the DOF of the various factors.

(2) Total sum of squares

(4) $$\mathrm{SSt}=\sum_{i=1}^{n}y_{i}{}^{2}-\frac{1}{n}{\left(\sum_{i=1}^{n}y_{i}\right)}^{2}$$

where $y_{i}$ is the S/N ratio of an experimental observation, and n is the total number of observations.

(3) Main effect of the sum of squares (SS)

If factor p has n levels and each level has m observed values, the sum of squares of factor p is expressed as:

(5) $${\mathrm{SS}}_{p}=\frac{\left(A_{1}{}^{2}{+A}_{2}{}^{2}+\ldots {+A}_{n}{}^{2}\right)}{m}-\mathrm{CF}$$

where $A_{i}$ is the value of the experimental observations for level i

(4) Error sum of squares

(6) $${\mathrm{SS}}_{E}={\mathrm{SS}}_{t}\;-\sum_{k=1}^{p}{\mathrm{SS}}_{p}$$

where p is the number of factors.

(5) Mean square and error mean of square:

(7) $$\mathrm{MS}=\frac{\mathrm{SSp}}{\mathrm{DOF}}$$

(8) $$\mathrm{MSE}=\frac{{\mathrm{SS}}_{E}}{{\mathrm{DOF}}_{E}}$$

(6) F-ratio

The F-ratio is defined as the mean square of the factor divided by the mean square of the pooled error.

(9) $$F=\frac{MS}{MSE}$$

(7) Partial sum of square ($SS^{\prime }$) of factors

(10) $${SS_{p}^{\prime }=\mathrm{SS}}_{P}-\mathrm{DOF}\;\times \;\mathrm{MSE}$$

(8) Percent of contribution

The percent of contribution is:

(11) $${\mathrm{CNp}=SS_{p}^{\prime }/\mathrm{SS}}_{t}$$

(9) Error

Error refers to the experimental error of the ANOVA assessment

(10) Combined error

It is the incorporation of insignificant factors in the analysis of variables into the error term, where insignificant factors are factors with lower contribution.

### 3.3. Confidence Interval (CI)

The confirmation experiment is performed to determine whether the experimental results and prediction results with a probability in a certain confidence interval. It can be employed to confirm whether the mathematical model built for the data from the orthogonal array experiments is suitable. The S/N ratio in the optimum condition (SN) can be predicted from the obtained optimum factor level setting value. The equation applied to compute SN is

(12) $$\hat{S}N=\bar{T}+\sum_{i=1}^{n}\left(F_{i}-\bar{T}\right)$$

where $\bar{T}$ is the general average of the S/N ratio; and F_i_ is the S/N ratio of significant factor level value i.

The equation used to compute CI is expressed as follows:

(13) $$CI=\sqrt{F_{\alpha ;1,V_{2}}\times M_{e}\times (\frac{1}{n_{eff}}+\frac{1}{r})}$$

where $F_{\alpha ;1,v_{2}}$ is the F value with significance level α; the confidence level is 1-α; $v_{2}$ is the DOF of pooled error mean square; $M_{e}$ is the pooled error mean square; r is the number of experiments; and $n_{eff}$ is the number of effective observations.

(14) $$n_{\mathrm{eff}}=\frac{n}{1+\left({\mathrm{DOF}}_{F_{i}}\right)}$$

where n is the total number of experiments, and ${\mathrm{DOF}}_{F_{i}}$ is the sum of degrees of freedom of significant factors.

The verification expression is used to validate the effectiveness of the predicted average.

(15) $$\hat{S}N-95\%CI\leq \mu \leq \hat{S}N+95\%CI$$

where μ is the true mean of the experiment.

### 3.4. Grey Relational Analysis (GRA)

The Taguchi method is a powerful optimization tool, but it is not suitable for the simultaneous optimization of multi-objective functions [38]. In general, the GRA model allows the simultaneous evaluation of different objective functions and enables the determination of the optimum parameters for all objective functions in the multi-response optimization problem [38,39,40]. Each quality of the output was individually optimized by the Taguchi method, and then all qualities were optimized together, taking into account the primacy of the targets by the Taguchi method and GRA. The relationship between the objective values and the sets of characteristics can be obtained in the execution experiments from orthogonal table. It is carried out as the following step:

(1) Target values of multiple quality items:

(16) $$\mathrm{Set}\;\mathrm{reference}\;\mathrm{sequence}\;X_{0}{=(x}_{0}{(1),x}_{0}{(2),x}_{0}{(3),x}_{0}{(4),x}_{0}\left(5)\right)$$

Multi-quality data sets obtained from the orthogonal table experiments for each group i:

(17) $$X_{i}=\left(x_{1}\left(1\right),\;x_{1}\left(2\right),\;x_{1}\left(3\right),\;x_{1}\left(4\right),\;x\left(5\right)\right),\;i=1,2,\ldots ,18.$$

The correlation between the objective value for quality k and the experimental observation value for k of group i is given by

(18) $$\gamma (x_{0}(k),x_{i}(k))=\frac{\zeta \underset{1\leq m\leq 5}{\max}\underset{k}{\max}\Delta_{0,m}(k)}{\Delta_{0,j}(k)+\zeta \underset{1\leq m\leq 5}{\max}\underset{k}{\max}\Delta_{0,m}(k)},k=1,2,\ldots ,5.,\;i=1,2,\;\ldots ,18.$$

which is called the correlation coefficient of X_i_ with X_0_ at point k.

This statistic represents the relationship of X_0_ with X_i_ for point k, indicating that it is a local condition. The mean of $\gamma (x_{0}(k),x_{i}(k))$ overall k is the correlation of X_i_ with X_0_.

(19) $$\gamma (X_{0},X_{i})=\frac{1}{n}\sum_{k=1}^{n}\gamma (x_{0}(k),x_{i}(k))$$

(2) The calculation steps for the gray relational grade:

Step 1: The initial value of each sequence is obtained.

(20) $$X_{i}^{\prime }=X_{i}/x_{i}(1)=(x_{i}^{\prime }(1),x_{i}^{\prime }(2),\cdots ,x_{i}^{\prime }(n)),\;i=0,\;1,\;2,\cdots ,m$$

Step 2: Obtain the differential sequence

(21) $$\Delta_{i}(k)=|x_{0}^{\prime }(k)-x_{i}^{\prime }(k)|,$$

(22) $$\Delta_{i}=(\Delta_{i}(1),\Delta_{i}(2),\cdots ,\Delta_{i}(n)),\;i=1,2,\cdots ,m$$

Step 3: Obtain the highest and lowest differences of both sides

(23) $$M=\underset{i}{\max}\underset{k}{\max}\Delta_{i}(k),\;m=\underset{i}{\min}\underset{k}{\min}\Delta_{i}(k)$$

Step 4: Obtain the relation coefficient

(24) $$\gamma_{0i}(k)=\frac{m+\zeta \;M}{\Delta_{i}(k)+\zeta },\;\zeta \in (0,1),\;k=1,2,\cdots ,n;\;i=1,2,\cdots ,m$$

Step 5: Obtain the relational grade

(25) $$\gamma =\frac{1}{n}\sum_{k=1}^{n}\gamma_{0i}(k),\;i=1,2,\cdots ,m$$

## 4. Experimental Process and Planning

### 4.1. Experimental Process

**Figure 4:**
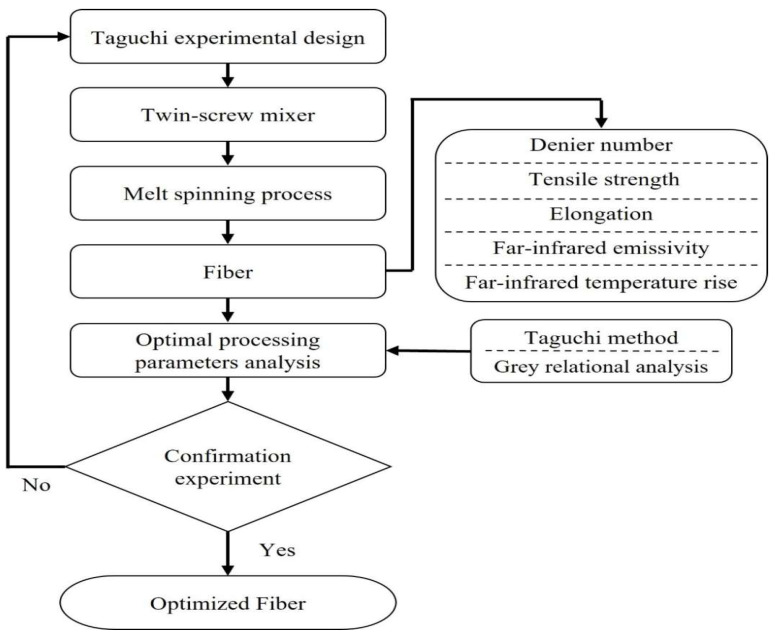
The flowchart of the optimized design process.

The graphene-modified polyester fiber in this study is made by adding nano-graphene powder into polyester particles, mixing the powder with twin screws to make the powder evenly dispersed, and then processing it into fibers by melt spinning. In this study, the Taguchi method was used to design experiments to find the best parameters for a single quality characteristic, and then combined with the grey correlation analysis method to find the best parameters to solve multiple quality problems and find the optimized fiber process parameters. The optimized design process is shown in Figure 4.

### 4.2. Melt Spinning Process Parameter Selection

Melt spinning process parameters are key factors affecting quality characteristics. Process parameters include nanographene content, melt temperature, mold temperature, gear pump speed, roller speed, and take-up speed; quality characteristics include yarn count, tensile strength, elongation at break, far-infrared emissivity and far-infrared temperature rise. The process parameters in this study were selected based on the following considerations:

(1) The nano-graphene powder addition

During the melt blending process of the twin-screw mixer, the polyester was pulverized, then melt-mixed with dispersant [41] and nanographene powder to improve the uniformity of the composite. The amount of nanographene powder added is based on the test results: fibers with a content ratio below 1.0 wt% have poor performance, and when the content ratio exceeds 2.0 wt%, yarn breakage occurs during melt spinning. In this experiment, the content of nanographene masterbatches obtained by biaxial processing was 1.0 wt%, 1.5 wt% and 2.0 wt%, respectively. That is: PET 97.5 wt%/1.5 wt% dispersant/1.0 wt% nanographene, PET97 wt%/1.5 wt% dispersant/1.5 wt% nanographene, PET 96.5 wt%/1.5 wt% dispersant/2.0% nanographene.

(2) Melt Spinning Temperature

Temperature below the pyrolysis temperature of the polyester (366 °C) was chosen. According to our experiments, the polyester fiber was selected as the basis for the spinning temperature in the melt spinning processing temperature range of 278~282 °C. Therefore, in terms of spinning process parameters, the melt spinning processing temperature of 278~282 °C is suitable for polyester materials.

(3) Gear pump speed

According to the experience value of the melt spinning machine, the speed of the gear pump for producing 75 denier fully drawn yarn is 13~17 rpm.

(4) Roller speed and take-up speed

According to the production of 75 denier processing yarn, the winding speed is 2300~2500 m/min, and the roller speed is higher than the take-up speed range of 40~60 m/min to improve the formation of yarn. Therefore, the speed of the roller is 2350~2550 m/min.

### 4.3. Taguchi Experiment Factor and Level Planning

In this study, spinning-grade PET and nanographene were melt-mixed through a twin-screw extruder, and 75 d/72 f processed yarn was obtained by melt spinning. Nanographene powder content, mold temperature, gear pump speed, melt temperature, roller speed and take up speed are used as control factors. The yarn count, tensile strength, percentage elongation, far-infrared emissivity and far-infrared temperature rise are taken as the quality characteristics. The planning experiments are shown in Table 1 and Table 2.

## 5. Results and Discussion 

Eighteen experiments were carried out through the experimental plan in Table 2 for each quality. The experiments were conducted three times to calculate the average value, standard deviation and S/N ratio.

### 5.1. Optimal Analysis of Yarn Count Single Quality

The yarn count of the fibers was tested according to ASTM D1577. The experiment data is shown in Table 3.

(1) Main effects analysis (MEA)

The response value of each factor at each level is calculated corresponding to the level of the orthogonal table. The maximum level of the response value is the highest level of single quality. The optimal parameters are shown in the response table of the yarn count in Table 4.

From Table 4, it can be seen that the best factor levels are A1, B3, C3, D3, E2 and F2, which have a nanographene powder content of 1%, mold temperature of 278 °C, gear pump speed of 17 rpm, melting temperature 282 °C, roller speed 2450 m/min and take-up speed of 2400 m/min. According to the influence of control factors, the order is gear pump speed > take-up speed > melting temperature > mold head temperature> roller speed > nanographene powder content.

(2) Analysis of variance (ANOVA)

The contribution of each factor to the quality characteristics was calculated through ANOVA. The ANOVA of the yarn count is shown in Table 5.

From Table 5, it is confirmed that the larger the F ratio, the greater the influencing factor. The factor that has the greatest influence on the yarn count is factor C (speed of the gear pump), followed by factor F (take-up speed), factor D (melting temperature), factor B (mold temperature), factor E (roller speed), and factor A (nanographene powder content).

(3) Yarn count confirmation experiment

Confirmation experiments were designed for the main control factors C3, D3 and F2, as shown in Table 6.

From Table 6, the 95% confidence interval was 36.75 ≤ µ ≤ 39.25. The experiment result of µ was 37.67. This shows that the optimized parameter combination obtained by the Taguchi method has good reproducibility.

### 5.2. Optimal Analysis of Tensile Strength Single Quality

The tensile strength of the fiber was tested according to ASTM D382. The experimental data were shown in Table 7.

(1) MEA

MEA was performed on the tensile strength data obtained from the experiments. The optimal parameters are shown in the response table in Table 8. 

From Table 8, it can be seen that the optimal factor levels are A3, B3, C3, D3, E2 and F3; that is, the nanographene powder content is 2%, the mold temperature is 281 °C, the gear pump speed is 17 rpm, the melt temperature is 282 °C, roller speed is 2450 m/min, and take-up speed is 2500 m/min. The controlling factors are take-up speed > roller speed > gear pump speed > mold temperature > nanographene powder content > melt temperature in descending order of influence.

(2) ANOVA

The contribution of each factor to the quality characteristics was calculated through the analysis of variance. The ANOVA of the tensile strength characteristics is shown in Table 9.

It can be observed from Table 9 that the most significant factor for tensile strength is factor F (take-up speed), followed by factor E (roller speed), factor C (gear pump speed), factor B (mold temperature), factor A (nanographene powder content), and factor D (melting temperature).

(3) Tensile strength confirmation experiment

Confirmation experiments were designed for the main control factors B3, C3, E2 and F3 using their optimal parameter levels as shown in Table 10.

From Table 10, the 95% confidence interval was 10.26 ≤ µ ≤ 11.14. The experiment result of µ was 10.53. This shows that the optimized parameter combination obtained by the Taguchi method has good reproducibility.

### 5.3. Optimal Analysis of Single Quality of Elongation at Break

The elongation at the break of the fiber was tested according to ASTM D382. The experimental data of elongation at break were shown in Table 11.

(1) MEA

The response table of the elongation at break is shown in Table 12.

From Table 12, the optimal factor levels are A3, B3, C2, D2, E1 and F1; that is, the nanographene powder content is 2%, the mold temperature is 281 °C, the gear pump speed is 15 rpm, and the melt temperature is 280 °C, roller speed 2350 m/min, and take-up speed 2300 m/min. The control factors are ranked in descending order of influence: take-up speed > roller speed > gear pump speed > nanographene powder content > melt temperature > mold temperature.

(2) ANOVA

The ANOVA of percentage elongation characteristics is shown in Table 13.

It can be observed from Table 13 that the most significant factor for tensile strength is factor F (take-up speed), followed by factor E (roller speed), factor C (gear pump speed), factor A (nanographene powder content), factor D (melting temperature), and factor B (mold temperature).

(3) Elongation at break confirmation test

Confirmation experiments were designed for the main control factors C2, E1 and F1 using their optimal parameter levels, as shown in Table 14.

As shown in Table 14, the 95% confidence interval was 28.41 ≤ µ ≤ 30.57. The experiment result of µ was 28.52. This shows that the optimized parameter combination obtained by the Taguchi method has good reproducibility.

### 5.4. Optimal Analysis of Single Quality of Far-Infrared Emissivity

The far-infrared emissivity of the fibers was tested according to the standard of FTTS-FA-010 [42]. The experimental data of far-infrared emissivity are shown in Table 15.

(1) MEA

The response table of far-infrared emissivity is presented in Table 16.

From Table 16, the optimal factor levels are A3, B3, C2, D2, E1 and F1; that is, the nanographene powder content is 2%, the mold temperature is 281 °C, the gear pump speed is 15 rpm, melting temperature is 280 °C, roller degree is 2350 m/min, and the take-up speed is 2300 m/min. The order of the controlling factors from the greatest to the least influence is nanographene powder content > take-up speed > mold temperature > roller speed > melting temperature > gear pump speed.

(2) ANOVA

The ANOVA of the far-infrared emissivity characteristics is presented in Table 17.

It is confirmed from Table 17 that the larger the F ratio is, the greater the influencing factor. The most significant controlling factor for the far-infrared emissivity is the factor A (nano-graphene powder content), followed by factor F (take-up speed), factor B (mold temperature), factor E (roller speed), factor D (melting temperature), and factor C (gear pump speed).

(3) Far-infrared emissivity confirmation experiment

Confirmation experiments were designed using their optimal parameter levels for the main control factors A3, B3, and F1, as shown in Table 18.

From Table 18, the 95% confidence interval was 38.34 ≤ µ ≤ 38.58. The experiment result of µ = 38.46. This shows that the optimized parameter combination obtained by the Taguchi method has good reproducibility.

### 5.5. Optimal Analysis of Single Quality of Far-Infrared Temperature Rise

The far-infrared temperature rise of the fibers was tested by the standard of FTTS-FA-010. In total, 18 experiments were carried out through the experimental plan, and the measurement was carried out three times to calculate the average value. The standard deviation and S/N ratio are shown in Table 19.

(1) MEA

The response table of the far-infrared temperature rise is shown in Table 20.

Table 20 presents that the best factor levels are A3, B2, C3, D3, E3 and F2, which are the nanographene powder content of 2%, the mold temperature of 278 °C, and the gear pump speed of 17 rpm, melting temperature 282 °C, roller speed 2550 m/min and take-up speed 2400 m/min. According to the influence degree of the control factors, the order is as follows: nanographene powder content > melting temperature > roller speed > gear pump speed > mold temperature > take-up speed.

(2) ANOVA

The ANOVA of the far-infrared temperature rise characteristics is shown in Table 21.

From Table 21, the most significant controlling factor for far-infrared temperature rise is A (content of nano-graphene powder), followed by factor D (melting temperature), factor E (roller speed), factor C (gear pump speed), factor B (mold temperature), and factor F (take-up speed).

(3) Far-infrared temperature rise confirmation experiment

The confirmation experiment was designed for the main control factor A3 using its optimal parameter level, as shown in Table 22.

From Table 22, the 95% confidence interval was 26.47 ≤ µ ≤ 27.15. The experiment result of µ was 26.73. This shows that the optimized parameter combination obtained by the Taguchi method has good reproducibility.

### 5.6. Multi-Quality Optimization

The research uses nanographene powder content, mold temperature, gear pump speed, melt temperature, roller speed and take-up speed as control factors, and uses yarn count, tensile strength, percentage elongation, distance infrared emissivity and far-infrared temperature rise as quality characteristics. The experiments designed from Taguchi’s method to provide the basis for a grey relational analysis that can formulate a multi-quality optimal parameter combination of fiber processing. The grey relational grade is obtained from GRA, as shown in Table 23.

The GRA transforms the five quality characteristics of the L18 orthogonal table into a grey relational grade, which is an indicator of how close the characteristics are to the reference sequence (37.97, 10.59, 30.49, 38.49, 26.91). The reference sequence is the maximum value of the SN value in the L18 orthogonal table of the five quality characteristics. A rank of 1 indicates complete overlap with the reference sequence. Therefore, the larger the correlation coefficient (r), the better. 

MEA was performed on the grey relational data obtained from all experiments; the response table is presented in Table 24.

From Table 24, the optimal factor levels are A3, B3, C3, D3, E2 and F1; that is, the nanographene powder content is 2%, the mold temperature is 281 °C, the gear pump speed is 17 rpm, the melting temperature is 282 °C, roller speed is 2450 m/min, and take-up speed is 2300 m/min. The order of the control factors from the greatest to the least influence is the nanographene powder content > gear pump speed > take-up speed > mold temperature > roller speed > melting temperature.

### 5.7. Confirmation Experiment

This research adopts the Taguchi method to design systematic experiments to optimize various parameters of graphene polyester fiber production. Comparing the optimal combinations in Table 25, showing the optimization parameters for a single quality, it can be seen that the optimal parameter combinations are different.

Therefore, in this study, the Taguchi method combined with GRA was used to convert five quality characteristics into grey correlation coefficients, and then quantify them into a single index (grey correlation, as shown in Table 23) to further obtain the optimal combination of multiple quality characteristics, that is, the nanographene powder content is 2%, the mold temperature is 281 °C, the gear pump speed is 17 rpm, the melting temperature is 282 °C, the roller speed is 2550 m/min, and the take-up speed is 2300 m/min. Finally, the confirmation experiment with 95% confidence interval as the standard verifies the conclusion that the combination is the optimal combination. A comparison of the properties of the best modified polyester yarns in this study with those of polyester yarns is shown in Table 26. 

It can be seen from Table 26 that the quality of the optimized modified polyester yarn is not only comparable to that of the unmodified polyester fiber, but also its functional far-infrared emissivity and far-infrared temperature rise are significantly improved.

Finally, the SN values of the five quality characteristics are all within the 95% confidence interval through confirmation experiments, respectively: the yarn count is 37.67 dB (the 95% confidence interval is between 36.75~39.25 dB), and the tensile strength is 10.53 dB (95% confidence interval is between 10.26~11.14 dB), the percentage elongation is 28.52 dB (95% confidence interval is between 28.41~30.57 dB), the far-infrared radiation rate is 38.46 dB (the confidence interval is between 38.34~38.58 dB), and the far-infrared temperature rise is 26.73 dB (the confidence interval is between 26.47 ≤ µ ≤ 27.15 dB), which means that the multi-quality optimization conditions obtained by the Taguchi method combined with the gray correlation analysis in this study are reliable.

The yarn count of the modified yarn optimized in this study is 76.5 d, which is 0.3 d higher than that of the unmodified polyester yarn of 76.2 d. Tensile strength of 3.3 g/d exceeds the industry standard of 3.0 g/d. Its percentage elongation is 26.7%, which is 3.2% higher than that of unmodified polyester yarn, which is 23.5%. These mechanical properties of the yarn were improved by experimentation with the production process parameters. The far-infrared emissivity of the optimized modified polyester yarn is 83%, which is 5.0% higher than that of the polyester yarn of 78%.

For far-infrared temperature rise measurement:

(1) Measuring instrument: 500-watt halogen lamp and infrared thermal phase detector.
(2) Test sample: sample cloth 5 × 5 cm.
(3) Test conditions and procedures: 500 W halogen lamp irradiated 100 cm away from the sample fabric, using infrared thermal imaging.

The thermometer takes a thermal image at 50 cm, continuously tests for 10 min at 1 min intervals, and records the far-infrared temperature rise of the fabric.

**Figure 5:**
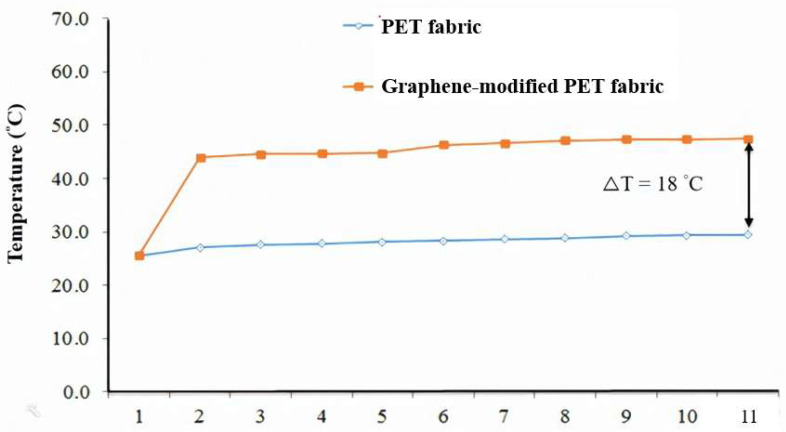
Far-infrared temperature rise curve of the optimized modified polyester yarn.

The far-infrared temperature rise in the modified optimize yarn in this study is 22.0 °C, which is 18.0 °C higher than that of regular polyester yarn of 4.0 °C from Figure 5. The regular polyester yarn does not add nano-graphene powder; the temperature rise is not obvious during the far-infrared temperature rise test.

### 5.8. Optimization of Thermal Properties and Surface Resistance of 75 d/72 f Yarns

The thermal properties and surface resistance of the optimized modified polyester yarns developed in this study are shown in Table 27.

In Table 27, based on DSC analysis (model: DSC 4000, Perkin Elmer, Massachusetts, MA, USA, test conditions—(i) the first stage of heating: 10 °C/min from 0 °C to 250 °C; (ii) the second stage of cooling: 3 °C/min from 250 °C to 0 °C; (iii) the third stage of heating: 10 °C/min from 0 °C to 250 °C)—showed that the crystallization temperature of the optimized modified ester yarn is higher than that of polyester, indicating that the addition of graphene nanopowder can increase the crystallization temperature of polyester. Based on TGA analysis (model: TA-Q500, TA Instruments, Delaware, USA, the test conditions are: the temperature range is 0~600 °C), it is shown that the thermal cracking temperature of the optimized modified polyester yarn is higher than that of polyester, which proves that the optimized modified polyester yarn has higher heat resistance. Based on surface resistance meter analysis, (model: ACL935, ACL Staticide, Chicago, IL, USA, test conditions: resistivity limits: 103–1012 ohms per square), it is shown that the surface resistance of the optimized modified yarn is lower than that of polyester, indicating that adding nanographene powder can reduce the static electricity of the fiber.

### 5.9. Fiber Surface Observation

**Figure 6:**
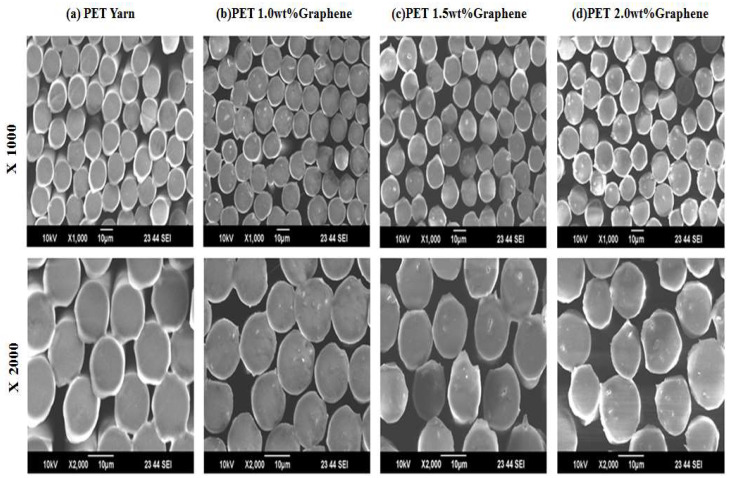
Fiber cross-section observation (**a**) conventional polyester yarn, (**b**) 1.0 wt% nanographene powder, (**c**) 1.5 wt% nanographene powder, (**d**) 2.0 wt% nanographene powder.

In this study, the scanning electron microscope (SEM, model: TM 3000, Hitachi, Tokyo, Japan Japan, test conditions: accelerating voltage 10 kV multiplier × 500 ~3000) was used to observe the particle size distribution of the cross-section of the modified yarn to which different proportions of nanographene powder were added, as shown in Figure 6.

It can be found from Figure 6 that when the content of nanographene powder increases, the particle distribution content in the fiber can also increase, which confirms that the nanographene powder in this study is indeed mixed with conventional polyester fibers.

### 5.10. Fourier-Transform Infrared (FTIR) Spectroscopy Analysis

**Figure 7:**
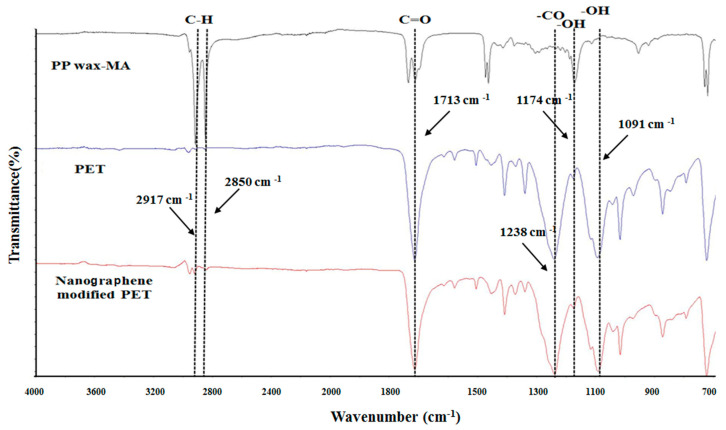
FTIR analysis result of various polymers.

The functional group analysis of PPwax-MA, PET, and nanographene-modified PET using FTIR (model: FTS 1000, Digilab, Inc., Hopkinton, MA, USA, test conditions: wavelength range of 400 to 4000 cm^−1^) was shown in Figure 7.

It was observed that the wavenumber of each polymer was the same, and the infrared transmittance was about 45–100%.

(1) It is observed that the carboxyl group has a characteristic absorption peak at the wavenumber of 1713 cm^−1^, indicating that the graphene-modified PET structure has a maleic anhydride structure C=O bond.
(2) The carboxyl group has a characteristic absorption peak at the wavenumber of 1238 cm^−1^, which represents the ether bond on the PET material.
(3) The characteristic absorption peaks of the hydroxyl group of the graphene structure were found at the wavenumbers of 1091 cm^−1^ and 1174 cm^−1^, indicating that the nano-graphene powder interacted with PPwax-MA/PET during the mixing process, increasing their compatibility.

## 6. Conclusions

In this study, we developed a Nano-graphene/polyester composite. The thermal insulation and performance of the polyester composite are enhanced as a function of far-infrared radiation, temperature rise, and antistatic. The Taguchi orthogonal array combined with gray correlation analysis (GRA) was used to optimize the process parameters of a single quality. The optimal formula combination is a nano-graphene powder content of 2%, mold temperature of 281 °C, gear pump speed of 17 rpm, melt temperature of 282 °C, hot roll speed of 2550 m/min, and take-up speed of 2300 m/min. The yarn count is 76.5 denier, 0.3 d higher than that of the unmodified polyester yarn of 76.2 d, and the tensile strength is 3.3 g/d, reaching the industry standard of 3.0 g/d. The elongation at break was 26.7%, which was 3.2% higher than the 23.5% of the unmodified polyester yarn. The far-infrared emission rate is 83%, which is 5.0% higher than the 78% of the unmodified polyester yarn. The far-infrared temperature rise is 22.0 °C, which is 18 °C higher than the 4.0 °C of the unmodified polyester yarn. Our findings have confirmed that the process-optimized yarns of the nanographene-modified polyester effectively improve the yarns’ mechanical properties and far-infrared functions. It can be used to develop multifunctional nano-graphene polyester fabrics for winter fabrics.

## Figures and Tables

**Table 1:** Optimal parameter design of graphene-modified polyester fiber.

Control Factor		Level		
		1	2	3
A	Graphene powder content (wt%)	1.0	1.5	2.0
B	Mold temperature (°C)	275	278	281
C	Gear pump speed (rpm)	13	15	17
D	Melt speed (°C)	278	280	282
E	Roller speed (m/min)	2350	2450	2550
F	Take up speed (m/min)	2300	2400	2500

**Table 2:** Optimization experiment plan of graphene-modified polyester fiber.

Exp. No.	Factors					
	A	B	C	D	E	F
	Graphene Powder Content (wt%)	Mold Temperature (°C)	Gear Pump Speed (rpm)	Melt Temperature (°C)	Roller Speed (m/min)	Take Up Speed (m/min)
1	1.0	275	13	278	2350	2300
2	1.0	278	15	280	2450	2400
3	1.0	281	17	282	2550	2500
4	1.5	275	13	280	2450	2500
5	1.5	278	15	282	2550	2300
6	1.5	281	17	278	2350	2400
7	2.0	275	15	278	2550	2400
8	2.0	278	17	280	2350	2500
9	2.0	281	13	282	2450	2300
10	1.0	275	17	282	2450	2400
11	1.0	278	13	278	2550	2500
12	1.0	281	15	280	2350	2300
13	1.5	275	15	282	2350	2500
14	1.5	278	17	278	2450	2300
15	1.5	281	13	280	2550	2400
16	2.0	275	17	280	2550	2300
17	2.0	278	13	282	2350	2400
18	2.0	281	15	278	2450	2500

**Table 3:** Experiment data of yarn count.

Exp. No.	Experiment				Yarn Count	
	Data 1 (d)	Data 2 (d)	Data 3 (d)	Mean (d)	Standard Deviation	S/N Ratio (dB)
1	75.3	74.5	75.0	74.9	0.40	37.49
2	79.5	76.8	81.6	79.3	2.40	37.97
3	78.1	77.5	80.6	78.7	1.64	37.91
4	74.2	72.3	74.6	73.7	1.22	37.34
5	73.5	71.5	84.2	76.4	6.82	37.60
6	77.8	75.3	80.3	77.8	2.50	37.81
7	76.1	75.1	78.1	76.4	1.52	37.66
8	73.1	71.8	78.2	74.4	3.38	37.41
9	74.4	80.7	76.0	77.0	3.27	37.71
10	78.8	77.2	76.8	77.6	1.05	37.79
11	70.3	68.9	74.5	71.2	2.91	37.03
12	75.1	75.5	82.0	77.5	3.93	37.77
13	75.0	77.0	77.9	76.6	1.48	37.69
14	77.5	76.8	80.0	78.1	1.68	37.85
15	75.5	75.1	77.5	76.0	1.28	37.61
16	76.7	77.5	78.6	77.6	0.95	37.79
17	76.8	77.5	78.2	77.5	0.70	37.78
18	72.1	70.8	77.7	73.5	3.66	37.30

**Table 4:** The response table of the yarn count (Unit: dB).

	A	B	C	D	E	F
Level 1	37.665	37.629	37.500	37.527	37.659	37.704
Level 2	37.651	37.609	37.666	37.653	37.666	37.774
Level 3	37.613	37.690	37.763	37.750	37.605	37.532
Effect	0.052	0.081	0.263	0.222	0.061	0.242
Ranking	6	4	1	3	5	2

**Table 5:** The ANOVA of the yarn count.

Factor	DOF	SS	MS	F Ratio	SS’	Contribution
A	2	0.0088	0.0044	0.1396	-	-
B	2	0.0216	0.0108	0.3408	-	-
C	2	0.2129	0.1064	3.3597	0.1495	19.5767
D	2	0.1497	0.0748	2.362	0.0864	11.3065
E	2	0.0133	0.0066	-	-	-
F	2	0.1990	0.0995	3.1396	0.1356	17.5148
Error	5	0.16	0.0316	-	-	-
Combined error	11	0.50	0.0454	-	0.39	51.602
Total	17	0.76	-	-	0.76	100

**Table 6:** The confirmation experiment of the yarn count (unit: Diner).

Confirmation Experiment	Unit: Diner				
Control factor	Data 1	Data 2	Data 3	Average	S/N
C3, D3, F2	77.3	75.5	76.8	76.5	37.67

**Table 7:** Experimental data of tensile strength.

Exp. No.	Experiment				Tensile Strength	
	Data 1 (g/d)	Data 2 (g/d)	Data 3 (g/d)	Mean (g/d)	Standard Deviation	S/N Ratio (dB)
1	3.16	3.12	3.13	3.14	0.021	9.92
2	3.22	3.28	3.26	3.25	0.030	10.24
3	3.42	3.35	3.39	3.39	0.035	10.59
4	3.28	3.30	3.26	3.28	0.02	10.31
5	3.19	3.15	3.22	3.19	0.035	10.06
6	3.29	3.17	3.31	3.26	0.075	10.25
7	3.17	3.25	3.19	3.20	0.041	10.11
8	3.28	3.31	3.34	3.31	0.030	10.39
9	3.36	3.28	3.37	3.34	0.049	10.46
10	3.32	3.17	3.33	3.27	0.089	10.29
11	3.35	3.39	3.34	3.36	0.026	10.52
12	3.12	3.18	3.08	3.13	0.050	9.90
13	3.32	3.27	3.35	3.31	0.040	10.40
14	3.33	3.34	3.41	3.36	0.043	10.52
15	3.35	3.27	3.36	3.33	0.049	10.44
16	3.29	3.26	3.29	3.28	0.017	10.32
17	3.25	3.36	3.27	3.29	0.058	10.35
18	3.35	3.41	3.36	3.37	0.032	10.56

**Table 8:** The response table of the tensile strength.

	A	B	C	D	E	F
Level 1	10.248	10.228	10.337	10.317	10.204	10.200
Level 2	10.333	10.351	10.214	10.268	10.401	10.281
Level 3	10.366	10.367	10.398	10.361	10.342	10.466
Effect	0.118	0.139	.0.181	0.093	0.196	0.266
Ranking	5	4	3	6	2	1

**Table 9:** The ANOVA of the tensile strength.

Factor	DOF	SS	MS	F Ratio	SS’	Contribution
A	2	0.0448		0.8840	-	-
B	2	0.0696	0.0224	1.3740	0.0189	2.6499
C	2	0.1033	0.0348	2.0385	0.0526	7.3585
D	2	0.0259	0.0516	0.5120	-	-
E	2	0.1216	0.0129	2.3993	0.0709	9.9148
F	2	0.2233	0.0608	4.4057	0.1726	24.1307
Error	5	0.1300	0.1116	-	-	-
Combined error	9	0.4208	0.0253	-	0.5729	55.9461
Total	17	0.72	0.0467	-	0.72	100

**Table 10:** The confirmation experiment of the tensile strength.

Confirmation Experiment	Unit: g/d				
Control factor	Data 1	Data 2	Data 3	Average	S/N
B3, C3, E2, F3	3.33	3.39	3.37	3.36	10.53

**Table 11:** Experimental data of elongation at break.

Exp. No.	Experiment				Elongation at Break	
	Data 1 (%)	Data 2 (%)	Data 3 (%)	Mean (%)	Standard Deviation	S/N Ratio (dB)
1	26.3	26.4	25.2	26.0	0.632	28.28
2	24.6	24.9	24.3	24.6	0.302	27.82
3	22.3	24.1	22.3	22.9	1.039	27.17
4	21.3	21.9	21.7	21.6	0.301	26.70
5	27.5	28.0	29.3	28.3	0.912	29.02
6	26.9	27.4	26.6	27.0	0.366	28.61
7	23.6	23.8	25.2	24.2	0.634	27.66
8	24.0	24.9	23.7	24.2	0.634	27.67
9	26.7	26.4	26.1	26.4	0.302	28.42
10	25.7	25.9	24.2	25.3	0.908	28.04
11	24.5	23.5	25.0	24.3	0.733	27.71
12	33.7	32.2	30.3	32.0	1.732	30.09
13	25.2	23.6	24.7	24.5	0.798	27.78
14	26.9	25.3	26.3	26.2	0.786	28.34
15	26.2	27.1	25.4	26.2	0.842	28.37
16	38.4	32.9	30.5	34.0	4.050	30.49
17	26.4	27.2	26.5	26.7	0.454	28.53
18	26.5	27.4	25.3	26.4	1.046	28.41

**Table 12:** The response table of the elongation at break.

	A	B	C	D	E	F
Level 1	28.181	28.157	27.999	28.166	28.490	29.105
Level 2	28.138	28.183	28.464	28.525	27.957	28.174
Level 3	28.534	28.513	28.390	28.162	28.406	28.174
Effect	0.395	0.356	0.465	0.362	0.532	1.529
Ranking	4	6	3	5	2	1

**Table 13:** The ANOVA of percentage elongation.

Factor	DOF	SS	MS	F Ratio	SS’	Contribution
A	2	0.5671	0.2835	0.9601	-	-
B	2	0.4739	0.2369	0.8023	-	-
C	2	0.7507	0.3753	1.2709	0.1600	1.3445
D	2	0.5201	0.2601	0.8806	-	-
E	2	0.9826	0.4913	1.6635	0.3919	3.2924
F	2	7.1329	3.5664	12.0759	6.5422	54.9578
Error	5	1.45	0.2902	-	-	-
Combined error	11	3.73	0.3386	-	5.0165	40.4053
Total	17	11.90	-	-	11.90	100

**Table 14:** The confirmation experiment of the elongation at break.

Confirmation Experiment	Unit: %				
Control factor	Data 1	Data 2	Data 3	Average	S/N
C2, E1, F1	26.7	27.2	26.2	26.7	28.52

**Table 15:** Experimental data of far-infrared emissivity.

Exp. No.	Experiment				Far-Infrared Emissivity	
	Data 1 (%)	Data 2 (%)	Data 3 (%)	Mean (%)	Standard Deviation	S/N Ratio (dB)
1	81.7	80.3	80.2	80.7	0.840	38.14
2	80.3	80.3	79.9	80.1	0.229	38.07
3	81.3	81.4	80.4	81.0	0.548	38.17
4	80.2	81.6	81.7	81.2	0.814	38.18
5	79.6	80.8	83.2	81.2	1.789	38.19
6	80.5	80.3	81.2	80.7	0.439	38.13
7	82.3	82.3	83.2	82.6	0.502	38.34
8	81.9	80.6	84.4	82.3	1.904	38.30
9	82.6	81.4	83.5	82.5	1.017	38.32
10	79.9	79.4	80.2	79.9	0.436	38.04
11	78.0	78.9	81.4	79.4	1.779	38.00
12	82.0	81.7	86.0	83.2	2.391	38.39
13	82.8	81.9	80.8	81.8	0.990	38.25
14	82.6	82.3	85.7	83.5	1.898	38.43
15	81.7	82.9	83.5	82.7	0.880	38.34
16	82.8	82.7	83.1	82.9	0.219	38.36
17	83.7	82.6	86.0	84.1	1.735	38.49
18	83.1	82.1	83.5	82.9	0.703	38.37

**Table 16:** The response table of the far-infrared emissivity.

	A	B	C	D	E	F
Level 1	38.138	38.223	38.248	38.235	38.286	38.308
Level 2	38.257	38.247	38.272	38.279	38.239	38.239
Level 3	38.366	38.291	38.242	38.247	38.236	38.214
Effect	0.228	0.068	0.030	0.044	0.050	0.093
Ranking	1	3	6	5	4	2

**Table 17:** The ANOVA of the far-infrared emissivity.

Factor	DOF	SS	MS	F Ratio	SS’	Contribution
A	2	0.1564	0.0782	14.9994	0.1460	59.7779
B	2	0.0145	0.0072	1.3934	0.0041	1.6801
C	2	0.0030	0.0015	0.2924	-	-
D	2	0.0062	0.0031	0.6005	-	-
E	2	0.0096	0.0048	0.9292	-	-
F	2	0.0282	0.0141	2.7039	0.0177	7.2758
Error	5	0.026	0.0052	-	-	-
Combined error	11	0.0451	0.0041	-	0.0764	31.2662
Total	17	0.24	-	-	0.24	100

**Table 18:** The confirmation experiment far-infrared emissivity.

Confirmation Experiment	Unit: %				
Control factor	Data 1	Data 2	Data 3	Average	S/N
A3, B3, F1	82.6	83.5	85.3	83.8	38.46

**Table 19:** Far-infrared temperature rise experimental data.

Exp. No.	Experiment Data				Far-Infrared Temperature Rise	
	F 1 (°C)	F 2 (°C)	F 3 (°C)	Mean (°C)	Standard Deviation	S/N Ratio (dB)
1	20.2	20.8	20.3	20.4	0.321	26.20
2	20.4	20.6	20.1	20.4	0.251	26.17
3	21.1	20.8	20.7	20.9	0.208	26.38
4	20.7	20.5	20.1	20.4	0.305	26.20
5	20.5	21.2	20.9	20.9	0.351	26.38
6	20.5	20.4	20.8	20.6	0.208	26.26
7	22.1	22.0	22.4	22.2	0.208	26.91
8	21.4	21.8	22.3	21.8	0.450	26.77
9	22.1	21.9	22.4	22.1	0.251	26.89
10	21.3	21.5	21.6	21.5	0.152	26.63
11	21.6	21.0	20.7	21.1	0.458	26.48
12	20.4	20.9	20.8	20.7	0.264	26.32
13	21.5	22.1	21.7	21.8	0.305	26.75
14	22.7	21.7	22.0	22.1	0.513	26.89
15	21.7	21.9	21.5	21.7	0.200	26.72
16	22.1	21.8	21.9	21.9	0.152	26.82
17	22.1	21.8	22.4	22.1	0.300	26.88
18	21.8	22.0	22.3	22.0	0.251	26.86

**Table 20:** The response table of the far-infrared temperature rise.

	A	B	C	D	E	F
Level 1	26.367	26.588	26.567	26.603	26.534	26.587
Level 2	26.538	26.601	26.568	26.504	26.612	26.600
Level 3	26.860	26.576	26.630	26.658	26.619	26.578
Effect	0.492	0.024	0.062	0.153	0.085	0.022
Ranking	1	5	4	2	3	6

**Table 21:** The ANOVA of the far-infrared temperature rise.

Factor	DOF	SS	MS	F Ratio	SS’	Contribution
A	2	0.7505	0.3752	4.0268	0.5641	42.2537
B	2	0.0018	0.0099	0.0099	-	-
C	2	0.0155	0.0078	0.0836	-	-
D	2	0.0725	0.0362	0.3893	-	-
E	2	0.0271	0.0135	0.1455	-	-
F	2	0.0015	0.0007	0.0081	-	-
Error	5	0.466	0.0931	-	-	-
Combined error	15	0.585	0.0389	-	0.771	57.7463
Total	17	1.34	-	-	1.408	100

**Table 22:** The confirmation experiment of the far-infrared temperature rise.

Confirmation Experiment	Experiment				
Control factor	Data 1	Data 2	Data 3	Average	S/N
A3	21.3	22.1	21.8	21.7	26.73

**Table 23:** Gray correlation grade of each group of experiments.

Exp. No.	$r_{i}$	Exp. No.	$r_{i}$	Exp.	$r_{i}$
1	0.4103	7	0.6079	13	0.5751
2	0.4902	8	0.5555	14	0.8358
3	0.6188	9	0.6769	15	0.6000
4	0.4188	10	0.5227	16	0.7484
5	0.4836	11	0.4732	17	0.7432
6	0.5035	12	0.5900	18	0.6724

**Table 24:** Response table of the grey relational grade.

	A	B	C	D	E	F
Level 1	0.5175	0.5472	0.5537	0.5838	0.5629	0.6242
Level 2	0.5695	0.5969	0.5669	0.5671	0.6028	0.5779
Level 3	0.6674	0.6103	0.6308	0.6034	0.5886	0.5523
Effect	0.1499	0.0631	0.0770	0.0363	0.0399	0.0719
Ranking	1	4	2	6	5	3

**Table 25:** The single quality optimization parameter combination.

	Parameter	Nanographene Powder Content (wt%)	Mold Temperature (°C)	Gear Pump Speed (rpm)	Melt Temperature (°C)	Roller Speed (m/min)	Take Up Speed (m/min)
Quality							
Yarn count (d)		1	278	17	282	2450	2400
Tensile strength (g/d)		2	281	17	282	2450	2500
Elongation at break (%)		2	281	15	280	2350	2300
Far-infrared emissivity (%)		2	281	15	280	2350	2300
Far-infrared temperature rise (°C)		2	278	17	282	2550	2400

**Table 26:** Comparison of optimized modified polyester yarns and polyester yarns.

Item	75 d/72 f Yarn			75 d/72 f Fabric	
	Diner (d)	Tensile Strength (g/d)	Percentage Elongation (%)	Far-Infrared Emissivity (%)	Far-Infrared Temperature Rise (°C)
Conventional polyester	76.2	3.6	23.5	78	4.0
Optimized Modified Polyester	76.5	3.3	26.7	83	22.0

**Table 27:** Optimize thermal properties, surface resistance of fabrics.

Test Sample	PET	PET +2.0 wt% Nanographene
Melting point (°C)	253.35	252.08
Crystallization temperature (°C)	203.41	220.59
Pyrolysis temperature (°C)	390.05	395.39
Weight loss 1% Pyrolysis temp. (°C)	366.0	367.43
surface resistance (Ω)	10^12^	3 × 10^8^

## Data Availability

Not applicable.
